# Mitochondria Biology in Reproductive Function

**DOI:** 10.3390/antiox11101978

**Published:** 2022-10-04

**Authors:** Carla Tatone, Giovanna Di Emidio

**Affiliations:** Department of Life, Health and Environmental Sciences, University of L’Aquila, 67100 L’Aquila, Italy

Mitochondria are multitasking organelles involved in the maintenance of cell homeostasis. By integrating signaling networks, mitochondria activate an adaptive response to stress and provide the energy necessary for cell and organism survival. Reproductive function is a high-energy-demanding process aimed at allowing safe transmission of DNA to subsequent generations. Starting from the assumption that mitochondria-based events regulate different aspects of reproductive function, the articles included in this Special Issue provide relevant insights about the mechanisms regulating the mitochondrial impact on female and male fertility. Overall, mitochondria have been revealed to be crucial in physiological and pathological pathways in gametes and reproductive tissues, and potential therapeutic attempts to restore mitochondrial function are proposed including antioxidants strategies and enhancement of NAD+ metabolism.

Many contributions to the present Special Issue have provided a deep comprehension of the role mitochondria play in sperm function. It clearly emerges that the role of sperm mitochondria is not limited to ATP production. Rather, sperm mitochondria are essential to modulating key cellular events related to maturation, such as DNA condensation in the epididymis [1], cell survival upon integration of pro-apoptotic and anti-apoptotic signals [2], or fertilization, by regulating ROS homeostasis for capacitation and acrosome reaction [1].

Concerning metabolic aspects, the mechanisms by which mitochondria operate in the male gamete represent an important issue. Although glycolysis should be considered as the preferred metabolic pathway for the energetic support of sperm motility in many species, the presence of specific metabolites in the female reproductive tract along with intracellular compartmentalization of metabolic enzymes still influences ATP metabolic sources [2,3]. As reported by Castellini et al. [2] who have shown a comprehensive biochemical overview of the origin of human sperm ROS, mitochondria represent the key cellular organelles in the interplay between ROS generation and intrinsic apoptosis-like events [2]. Chianese and Pierantoni [3] have focused on the strict link between ROS and sperm functionality, pointing out that low levels of ROS are normally produced by human spermatozoa and are involved in sperm physiological processes. When the balance between ROS levels and antioxidant defense is perturbed, the derived oxidative stress harms the sperm at genomic, epigenomic, lipidomic, and proteomic levels. In accordance, the comparison of proteomic and transcriptomic profiles in fertile, sub-fertile, and infertile spermatozoa in varied mammalian species (bovine, porcine, and human species) suggests that oxidative damage can be ascribed to defects of mitochondrial energy metabolism-related proteins [1].

Moreover, as thoroughly reviewed by Chianese and Pierantoni [3], oxidative stress represents the mechanism by which paternal experience may influence the embryo development. Moreover, as a result of toxicants and xenobiotics interactions, through the paternal transgenerational inheritance, oxidative stress-induced sperm epimutations can be responsible for negative effects on the health of the offspring. Accordingly, the transgenerational study by Oliveira et al. [4] on the rat model evidenced that parabens administered to pregnant rat induced mitochondrial dysfunctions in testicles of the F1 generation. This event was accompanied by increased ROS production, as well as altered modulation of the antioxidant system in testes and other organs [4]. This is relevant since testis mitochondria are essential for spermatogonial stem cell differentiation, testicular somatic cell development, and testosterone production [1]. Therefore, sperm and testis mitochondrial health link extrinsic and intrinsic factors to fertility, influencing fertility potential and consequent embryo development, with possible deleterious effects on the offspring through the paternal transgenerational inheritance. An emerging role in counteracting imbalance of ROS and promoting antioxidant defenses in testes seems to be played by mitochondrial sirtuins (mtSIRTs), in particular by SIRT3 [5].

In order to investigate the effects of mitochondria-centered antioxidant approaches on the improvements of sperm functions and male reproductive potential, Ferramosca and colleagues [6] tested the effects of several plant polyphenols. By focusing on mitochondrial respiration efficiency measured by polarographic assays, the authors demonstrated that quercetin, naringenin, genistein, apigenin, luteolin, and resveratrol modulated sperm mitochondrial function by different mechanisms. In particular, quercetin, genistein, and luteolin were found to be effective in improving sperm mitochondrial function in asthenozoospermic samples [6].

Evidence from animal models revealed the interest of researchers in finding new parameters that may correlate mitochondria characteristics to sperm quality. By using high-precision new generation flow cytometry, Kern and colleagues [7] proposed that the length of boar sperm mitochondrial sheath, measured by using an aggresome marker, may represent a measurable sperm phenotype correlated with fertility. In the bovine model, Madeja et al. [8] discovered a correlation between the number and the activity of mitochondria and global sperm quality, by comparing sperm samples from bulls with high reproductive performance with samples from bulls temporarily disqualified from semen production. In this paper many mitochondrial parameters, including mtDNA copies, mitochondrial membrane potential, transcript-level of antioxidant enzymes, were correlated with sperm quality, although no differences were noted when samples were tested for oxygen consumption rate [8].

The strict link between maternal nutrition and oocyte mitochondrial activity is a relevant topic in the present Special Issue. In particular, Fabozzi et al. [9] highlighted the importance of healthy dietary habits to improve chances of conception and overall live birth rate. From animal models, it clearly emerges that unbalanced dietary intakes, such as a high-fat diet, high-fat and high-sugar diets, or low protein diet, result in overall negative effects on oocyte mitochondria in terms of intracellular distribution, content, structure, biogenesis, and functioning [9]. Nevertheless, the underlying mechanisms implicated in mitochondrial changes are not fully understood [9]. This lack of information is also evidenced by Rodriguez-Varela and Labarta [10], whose work focused on the efficacy of antioxidants including resveratrol, coenzyme Q10, melatonin, folic acid and vitamins in IVF clinical settings. The same authors pointed out that, although the current literature claims that these molecules act by improving mitochondrial function, direct evidence of these effects is lacking. Moreover, randomized control trials are required to overcome uncertainties regarding dose and duration of clinical application of antioxidant strategies [10]. The importance of proper antioxidant supplementation is further supported by data obtained by Moreira-Pinto et al. [11], who show that different concentrations of resveratrol may have contrasting effects on granulosa cells (GCs). Indeed, the authors demonstrated that, although ATP production and membrane mitochondrial potential remained unaffected by any concentration, low resveratrol concentrations have a protective role on GCs by reducing reactive species formation after stress induction, while high concentrations negatively affect GC viability and steroidogenic function [11]. Interestingly, Battaglia et al. [12] demonstrated that oral administration of resveratrol for 3 months prior to IVF can have beneficial effects on oocyte competence in reproductively aged women. This effect was correlated with changes in the expression profile of miRNAs contained in follicular fluids. Among differentially expressed miRNAs in the resveratrol group, miR-125b-5p, miR-132-3p, miR-19a-3p, miR-30a-5p and miR-660-5p were found to be involved in the regulation of mitochondrial proteins, controlling mitochondrial metabolisms and biogenesis, and referred to as mito-miRNA. In particular, Battaglia et al. [12] suggested that downregulation of miR-132 in follicular fluid of older women after resveratrol treatment could be relevant for oocyte metabolism since it may regulate carnitine-acyl-carnitine translocase. This enzyme drives the transport of acyl-L-carnitines to the inner mitochondrial membrane, where they enter the β-oxidation process, suggesting that beneficial effects of resveratrol are mediated by increased mitochondrial lipid metabolism in the aged ovarian microenvironment. As reported by Placidi and colleagues [13], acyl-L-carnitines activate antiapoptotic, antiglycative, antioxidant, and anti-inflammatory signaling in the female germ cell and reproductive system. Indeed, these authors highlighted the importance of acyl L-carnitine and the mechanisms by which these molecules may represent promising culture medium supplements to counteract altered energy and redox homeostasis associated with oocyte and embryo in vitro culture and cryopreservation in ART [13]. These reviews further sustain the hypothesis that mitochondria are the main targets of carnitine supplementation in PCOS mice diet, probably due to the activation of a SIRT1 pathway [5,14,15]. This is not surprising, since miR-132 is known to be deregulated with aging and stressing conditions in the mammalian oocyte, and Sirt1 is one of the most studied targets of its activity [16]. Therefore, resveratrol may improve follicular microenvironment by transcriptomic and proteomic modifications in granulosa cells [12], positively affecting oocyte competence by regulating lipid utilization and energetic metabolism [13]. Nevertheless, granulosa/cumulus cells are not always necessary for proper oocyte functioning and metabolism. Indeed, despite germline deletion of Clpp (caseinolytic peptidase P), the factor that promotes the degradation of unfolded mitochondrial proteins (UPRmt), resulting in female infertility and accelerated loss of ovarian reserve in mice, Esencan et al. [17] demonstrated that granulosa/cumulus cell-specific deletion of Clpp did not induce alterations in female fertility.

In the attempt to improve the effects of aging on oocyte quality and female fertility, interesting data have been provided by Min et al. [18] in Caenorhabditis elegans. When the nematode growth medium was supplemented with nicotinamide (NAM), a reduced mitochondrial ROS production and the amelioration of mitochondrial function were observed. These effects were also accompanied by decreased embryonic lethality and increased developmental growth and offspring motility [18]. Overall, this contribution supports the evidence that deregulation of NAD+ metabolism contributes to the aging phenotype and requires deeper understanding to provide potential anti-aging approaches. A cross-link between NAD+ metabolism and mitochondrial function is represented by mitochondrial sirtuins (mtSIRTs). Sirtuins are NAD+-dependent enzymes with a significant role in both the formation and the course of many gynecological diseases and are involved in some gynecological disturbances as regulative factors in pathways associated with insulin resistance, glucose and lipid metabolism disorders [19]. MtSIRTs, which are sirtuins located in the mitochondria, catalyze the deacylation and the ADP-ribosylation of mitochondrial proteins [5,15]. Interest on the role mtSIRTs in reproduction has been increasing in recent years, although most research is focused on animal models with very little information from human cells [5]. From the literature it emerges that the most studied mtSIRT in this field is SIRT3. This sirtuin has been strictly related to the acquisition of oocyte competence and protection against stress conditions in oocytes and embryos [5].

It is well known that embryos rely on oocyte mitochondrial content and functioning to face changes in energetic metabolism during key steps of early embryonic development [20]. During tubal–uterine transport, the embryo goes through different environments with different compositions and conditions, being exposed to a decreasing oxygen gradient while simultaneously shifting from an oxidative metabolism to a glycolytic one. The redox state is finely regulated during embryo development by maintaining the equilibrium of the most important redox couples. The authors point out that, at the cleavage stage, mitochondria have the role of maintaining the redox homeostasis and producing intermediate metabolites that are essential to cell signaling pathways and epigenetic reprogramming. Given that any mitochondrial dysfunction or excessive functioning can cause a perturbation in embryo development, a low mitochondrial activity would favor a “quiet metabolism” or, more specifically, a balanced and parsimonious metabolism [20].

To date, the main challenge is the identification of reliable mitochondrial markers that may drive the development of targeted mitochondria therapies in infertility. Recently, growing amounts of evidence are pointing to the study of qualitative and quantitative mitochondrial genome (mtDNA) alterations caused by oxidative stress as possible markers of reproductive outcomes [21]. A reduced copy number of oocyte mtDNA is related to fertilization failure and ovarian insufficiency. Correlation between mtDNA content in cumulus cells and in peripheral blood cells has been reported, and lower mtDNA copy number is observed in peripheral blood in females with a reduced ovarian reserve or ovarian insufficiency. Thus, the quantitative and qualitative mtDNA variations observed in ovarian, embryonic, and peripheral blood cells represent promising tools for predicting female fertility [21].

In the field of reproductive dysfunctions, endometriosis is a disease defined as the abnormal presence of endometrial-like tissue outside the uterus with negative effects on fertility. To enhance treatment methods for endometriosis, Song et al. [22] have searched for anti-proliferative effects of a flavonoid with anti-cancer effects and have found that 6,8-diprenylorobol affects cell proliferative pathways by inducing malfunction of the mitochondria, including loss of mitochondrial membrane potential, cellular respiration, and energy production in two different endometriotic cell lines. All these results indicated the therapeutic potential of 6,8-diprenylorobol in human endometriosis and will help to stimulate further research into the peculiar role of mitochondria in this disease [22].

One of the most relevant topics in the reproductive field is cryopreservation, a fundamental technology in Assisted Reproductive Techniques. Gualtieri et al. [23] has provided an overview based on a thorough and critical analysis of the literature. A close relationship between mitochondrial damage and ROS generation during cryopreservation of gametes and gonadal tissues has been well established in different species. Following a long evaluation of different mechanisms and hypotheses, Gualtieri et al. [23] propose the Ca^2+^ overload induced by permeating cryoprotectants as a potential trigger of mitochondrial damage associated with cryopreservation. This is followed by prolonged openings of mitochondrial permeability transition pores, increased ROS generation and cytochrome c release. The authors reported that a variety of antioxidants have been administered in different steps of freezing/thawing to prevent or minimize mitochondrial malfunction and oxidative stress and increase the reproductive capacity. However, the risk for a cytoplasmic reductive stress related to the use of antioxidants requires further investigation, as proper amounts of mitochondrial ROS are necessary to activate signaling pathways underlying reproductive cell functions.

In conclusion, this Special Issue brings together current findings concerning mitochondrial activities involved in reproductive function under normal and diseased states, as well as potential strategies to mitigate mitochondrial dysfunction, and provides a useful resource to stimulate further work in this fascinating area.
